# Thermodynamic Behavior of Doped Graphene: Impact of Heavy Dopant Atoms

**DOI:** 10.3390/e26121093

**Published:** 2024-12-14

**Authors:** L. Palma-Chilla, Juan A. Lazzús

**Affiliations:** 1 Departamento de Física, Universidad de La Serena, Casilla 554, La Serena 1700000, Chile; 2 Instituto de Investigación Multidisciplinario en Ciencias y Tecnología, Universidad de La Serena, Casilla 554, La Serena 1700000, Chile

**Keywords:** graphene, phase transitions, Gibbs entropy, Helmholtz free energy, numerical calculations

## Abstract

This study investigates the effect of incorporating heavy dopant atoms on the topological transitions in the energy spectrum of graphene, as well as on its thermodynamic properties. A tight-binding model is employed that incorporates a lattice composition parameter associated with the dopant’s effect to obtain the electronic spectrum of graphene. Thus, the substitutional atoms in the lattice impact the electronic structure of graphene by altering the connectivity of the Dirac cones and the symmetry of the energy surface in their spectrum. The Gibbs entropy is numerically calculated from the energy surface of the electronic spectrum, and other thermodynamic properties, such as temperature, specific heat, and Helmholtz free energy, are derived from theoretical principles. The results show that topological changes induced by the heavy dopant atoms in the graphene lattice significantly affect its electronic structure and thermodynamic properties, leading to observable changes in the distances between Dirac cones, the range of the energy spectrum, entropy, positive and negative temperatures, divergences in specific heat, and instabilities within the system.

## 1. Introduction

Graphene is a carbon structure characterized by a single layer of atoms arranged in a two-dimensional honeycomb pattern [1]. Each carbon atom in graphene forms bonds with three neighboring carbon atoms, creating a hexagonal lattice. This unique atomic arrangement gives graphene exceptional properties, making it a significant material in diverse scientific and technological fields [2].

The inert and gapless nature of graphene can be altered by introducing heavy dopant atoms that replace carbon atoms in the lattice [3]. These dopant atoms, which vary in size and electronic properties, create unique bonding configurations within the graphene structure. This substitution not only alters the electronic band structure but also introduces new topological features and enhances the material’s functionality [4,5,6]. Extensive theoretical and experimental studies have shown that doping with heavy atoms can lead to substantial changes in graphene’s physical and chemical properties, including its electronic, optical, and thermodynamic behaviors [7,8,9]. As a result, doped graphene, particularly with heavy dopants, emerges as a versatile material for a wide range of applications, including electronics [10], sensors [11], fuel cells [12], batteries [13], and water splitting [14]. The specific nature of each heavy dopant can tailor the material’s properties, allowing for enhanced performance and novel functionalities in various technological fields [15].

In addition, recent studies on graphene doped with heavy atoms have explored superconductivity under certain conditions. A theoretical approach has demonstrated that heavy doping with nitrogen (N) or boron (B) through the intercalation of 50% N or B atoms and stabilization under strain can yield high-temperature superconducting behavior in graphene [16]. Other instances of superconductivity in doped graphene include lithium-decorated and calcium-decorated graphene, both of which exhibit superconducting behavior at relatively high temperatures. In both cases, superconductivity is attributed to enhanced electron–phonon coupling from the heavy dopants, facilitating phonon-mediated superconductivity [17,18,19].

In this contribution, we utilize the nearest-neighbor tight-binding model to introduce a variation in one of the hopping parameters, assuming two different local wave functions. This enables us to model the compositional variation of the lattice in doped graphene. Through this approach, we explore the energy surface topology and thermodynamics by numerically solving the electronic spectrum. The objective of our research is to analyze how incorporating heavy dopant atoms affects topological transitions in the energy spectrum and the thermodynamic properties of graphene, in contrast to pristine graphene. Building on our previous work, we extend these studies [20,21] by applying their methodology to doped graphene with heavy atoms. To the best of our knowledge, there is no comparable application of these doped graphenes as proposed in this study.

## 2. Electronic Spectrum for Doped Graphene

**Figure 1:**
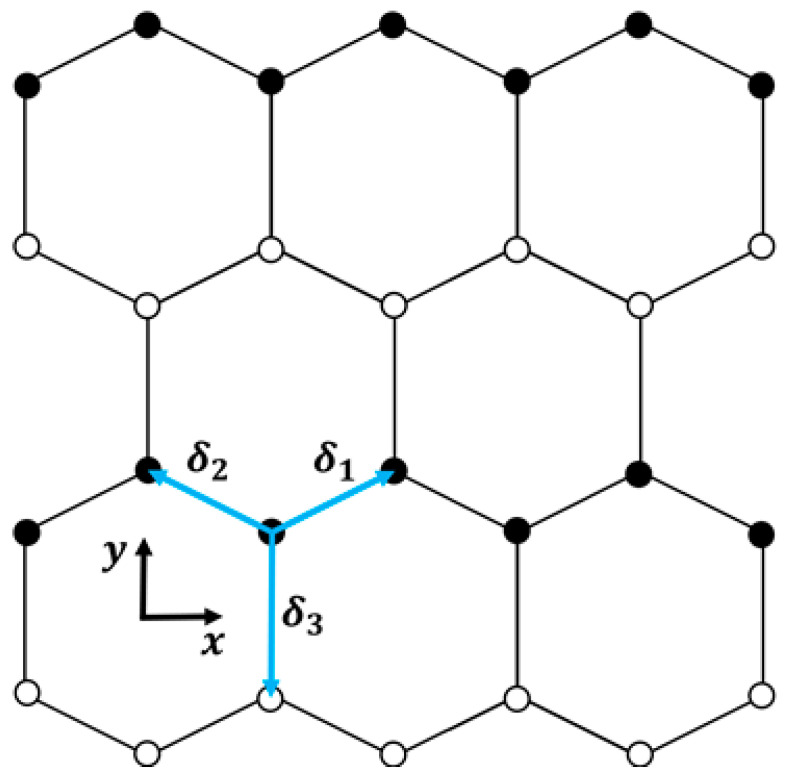
Representation of the hexagonal lattice of doped graphene used in this study. The illustration includes the three vectors $\delta_{1}$, $\delta_{2}$, and $\delta_{3}$, connecting each site to its nearest neighbors.

**Figure 2:**
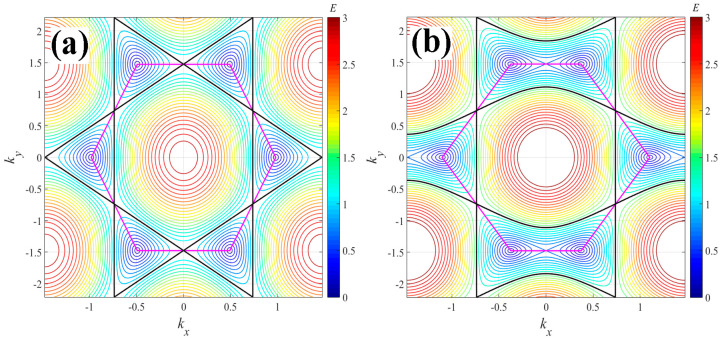
The energy spectrum of graphene represented as a contour map of the surface energy obtained by Equation (3): (**a**) pristine case γ=1: at E=1, it exhibits a topological transition when the energy levels pass from the six Dirac cones (lower energy levels) to only one central figure resembling a dome at upper energy levels (see black line). Additionally, a magenta line connects the Dirac cones of the cell; and (**b**) doped case γ=1.5 (1<γ<2): the topological transition occurs at E~1.5 (marked by the black line), and the Dirac cones shift in $k_{x}$ due to the compositional variation (observe changes in the magenta line compared to panel a). If γ≥2, the cones merge.

In the context of a two-dimensional honeycomb lattice illustrated in Figure 1, the tight-binding model can be derived from a hexagonal Bravais lattice. This lattice is characterized by the elementary vectors $a_{1}$ and $a_{2}$, along with the position vectors of the first-nearest neighbors $\delta_{1}$, $\delta_{2}$, and $\delta_{3}$, defined as follows:

(1) $$a_{1}=\frac{a}{2}\;\left(\sqrt{3},3\right)\;;\;a_{2}=\frac{a}{2}\;\left(-\sqrt{3},3\right)$$

(2) $$\delta_{1}=\frac{a}{2}\;\left(\sqrt{3},1\right);\;\delta_{2}=\frac{a}{2}\;\left(-\sqrt{3},1\right);\;\delta_{3}=\frac{a}{2}\;\left(0,-1\right)$$

where a represents the honeycomb lattice parameter, signifying the distance between atoms. To clarify, the coordinate system xy is established with the “x” axis aligned with the zigzag direction while “y” is aligned with the armchair direction in the lattice (see Figure 1). Moreover, assuming the hopping parameters as $t_{1}\left(\delta_{1}\right)=t_{2}\left(\delta_{2}\right)=t$ and $t_{3}\left(\delta_{3}\right)=\gamma t$, the electronic spectrum for a doped graphene results in the following:

(3) $$E\left(k_{x},k_{y},\gamma \right)=+t\sqrt{2+\gamma^{2}+4\gamma cos\left(\frac{\sqrt{3}}{2}ak_{x}\right)cos\left(\frac{3}{2}ak_{y}\right)+2cos\left(\sqrt{3}ak_{x}\right)}$$

where t (assumed unit) is the hopping term between the nearest atoms, and $k_{x}$ and $k_{y}$ are wavevector components along the x and y directions, respectively. Note that this model represents the one-electron energy spectrum within the upper band and does not account for transitions between bands. The parameter γ represents the ratio of the hopping energies between the nearest neighbor atoms in the hexagonal doped graphene lattice (e.g., $\gamma =t_{3}/t_{1}$). We consider that the presence of different atoms (with distinct γ values) in the doped graphene lattice produces different topologies on the surface of the energy spectrum and, consequently, leads to varied thermodynamic behaviors. Here, we study the cases where the hopping energy of the dopant atom is greater than that of the carbon atom, i.e., γ>1, where the dopant is an atom heavier than carbon (such as N, Si, among others). Crucially, to maximize the likelihood of achieving γ>1, it is essential that the dopant and carbon atoms are arranged alternately within the hexagonal cell structure (see Figure 1). If γ=1, Equation (3) reproduces the spectrum of pristine graphene (refer to Figure 2a). But, when 1<γ<2, the incorporation of heavy dopant atoms causes the Dirac cones to shift from the corners of the first Brillouin zone. If γ≥2, this results in the merging of two Dirac cones (see Figure 2b). As is known, graphene has a honeycomb structure with two sublattices; however, when some heavy dopant atoms are introduced, resulting in the case where γ≥2, they interact differently with the carbon atoms in each sublattice, causing a merging of the Dirac cones and leading to distortions in the graphene lattice, such as changes in bond lengths or bond angles. Due to this, we consider that this new lattice does not represent conventional graphene or one that can be accurately modeled with the tight-binding approach proposed in this study. Therefore, we will only focus on the thermodynamics of the 1<γ<2 cases, where the dopant atom is only slightly heavier than the carbon atom and does not break the symmetry of the lattice. Note that the case where γ<1 implies the use of dopant atoms lighter than carbon, where the hopping energy of these atoms is lower than that of carbon; therefore, this case will not be considered in this study. An analysis of the thermodynamic behavior of graphene doped with γ<1 can be found in [21].

Additionally, the model described in Equation (3) only characterizes graphene doped through substitutional atoms, so it cannot be applied to graphene doped with adatoms or other types that do not correspond to a hexagonal 2D lattice structure with six atoms per unit cell (see Figure 1). The model also considers that any structural distortion caused by the dopant is small enough not to disrupt the overall symmetry of the hexagonal graphene lattice. In other words, the substitutional dopant atoms integrate without significantly distorting the lattice, allowing for variations in $t_{3}/t_{1}$ without altering their 2D structure. In addition, varying the parameter $\gamma =t_{3}/t_{1}$ represents the consideration of different types of atoms as dopants. Note that an increase in γ implies a higher hopping energy $t_{3}$ between distinct atoms (carbon/dopant). Moreover, the periodicity in our model assumes that the dopant atoms are uniformly distributed across the graphene lattice, representing a highly doped lattice. It is worth noting that studies have predicted that, if a two-dimensional honeycomb graphene lattice is heavily doped, it will exhibit numerous fascinating properties, aligning our model with these theoretical predictions, e.g., [16,22,23,24].

## 3. Numerical Calculations and Thermodynamic Properties

To characterize the thermodynamic behavior of doped graphene, we adopt the methodology proposed by Flores [20]. This approach involves utilizing the electronic band structure graph of graphene (via Equation (3)) along with a horizontal plane intersecting it at a specified energy level, thus determining the points where both curves intersect. Subsequently, the numerical calculation determines the area enclosed by the intersection points in the horizontal cutting plane. The algorithm designed to perform these calculations consists of the following steps:

(1) *The numerical construction of energy surface:* construct the energy surface of graphene numerically based on the electronic spectrum equation (Equation (3));
(2) *The selection of energy level*: choose a specific energy level to establish a corresponding horizontal energy-cutting plane;
(3) *The identification of intersection curve*: identify the intersection curve formed by the energy surface and the selected energy-cutting plane;
(4) *The calculation of intersection area*: use the intersection points between both curves to calculate the intersection area, employing the Shoelace formula [25,26].

The algorithm iterates through these steps, enabling the calculation of the intersection area at various energy values. Specifically, 300 level sets within the energy interval $0<E\leq E_{max}$ are considered.

We assume that the number of states $\Omega \left(E\right)$ [27,28,29,30] is directly proportional to area $A\left(E\right)$ formed by the intersection of the energy-cutting plane with the energy spectrum of doped graphene (i.e., $A\left(E\right)\sim \Omega \left(E\right)$). Our approach implies that the degeneracy of the one-electron energy spectrum directly corresponds to the degeneracy of the system. Subsequently, we apply the Gibbs entropy $S_{G}$ [27,28,29,30] over the area using the following expression:

(4) $$S_{G}\left(A\left(E\right)\right)=k_{B}\ln\left(A\left(E\right)\right)$$

where $k_{B}$ is the Boltzmann constant. Note that we equate the number of states to the size of the Fermi surface, specifically the two-dimensional Fermi surface [21,27]. After obtaining the entropy, the remaining thermodynamic properties are numerically determined using the following relationships:

(5) $$T={\left(\partial E/\partial S_{G}\right)}_{V}$$

(6) $$C={\left(\partial E/\partial T\right)}_{V}$$

(7) $$F=E-TS_{G}$$

where T is the temperature, C is the specific heat, and F is the Helmholtz free energy. The theoretical foundations of these thermodynamic relations can be found in [27,28,29,30].

## 4. Compositional Effects on Thermodynamic Behavior

**Figure 3:**
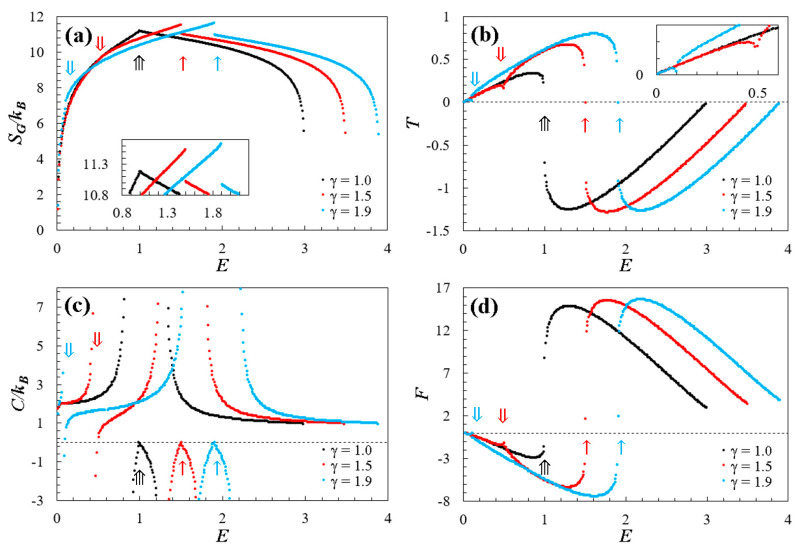
Thermodynamic properties (expressed by Equations (4)–(7)) are plotted against dimensionless energy E. In all panels, the doped cases γ=1.5 (red dots) and γ=1.9 (blue dots) are contrasted with the thermodynamics behavior of a pristine graphene γ=1 (black dots) [20,21]. The single (↑), double (⇓), and triple (⇛) arrows highlight identical points of interest for energy and its progression in each property. The panels represent (**a**) entropy, (**b**) temperature, (**c**) specific heat, and (**d**) Helmholtz free energy.

Using the methodology described above, we obtained numerical results for doped graphene subjected to compositional variations in its lattice with 1<γ<2. The outcomes for the investigated thermodynamic properties (Equations (4)–(7)) are illustrated in Figure 3. For a clearer comparison, we have selected the cases γ=1.5 and γ=1.9 to analyze the thermodynamic behavior of the doped graphene and contrast it with the behavior of pristine graphene (γ=1).

### 4.1. Entropy

Figure 3a shows the Gibbs entropy $S_{G}$ as a function of dimensionless energy E. Notably, the dopant significantly impacts the spectrum curve over the range $0<E<E_{max}$, with $E_{max}\propto \gamma$. This is well-fitted by the linear relation $E_{max}=\gamma +2$, with a correlation coefficient $R^{2}$ of 1. This influence extends to the maximum entropy $S_{G,max}\propto \gamma$, as depicted in the inner panel of Figure 3a. The maximum entropy can be fitted with a quadratic relation, specifically resulting in $S_{G,max}=-0.392\gamma^{2}+1.534\gamma +10.120$, with $R^{2}=0.996$ for 1<γ<2.

Within the low-energy spectrum zone (0<E≤~1) for doped graphene, a distinctive point of slope change is evident, denoted by a double arrow (⇓). Interestingly, this energy point aligns with the level where two Dirac cones merge due to the effect of the dopant, signifying a topological change in the low-energy regime (see Figure 3b,c for reference). For energies below the ⇓ point, entropy curves fit at the equation $S_{G}/k_{B}\sim 2\ln\left(E\right)$ [20,21], reflecting the equipartition principle [20,21,22,23]. For energies exceeding the ⇓ value, the entropy curves exhibit quasi-linear behavior, extending to a point marked by a single arrow (↑) when an entropy gap proportional to the compositional variation appears (${∆S}_{G,gap}\propto \gamma$). This can be fitted using the quadratic equation ${∆S}_{G,gap}=-0.546\gamma^{2}+2.176\gamma -1.517$, with $R^{2}=0.997$. Moreover, the energy at the specific point ↑ corresponds to the energy level on the spectrum’s surface where the topological transition occurs, transitioning from Dirac cones in the low-energy regime to “the spectrum dome” in the high-energy regime (see energy levels highlighted in black in Figure 3b,c). Note that the energy values at which both the energy regime change and the topological change occur ($E=E_{↑}$) are directly equivalent to the value of the compositional parameter, where $E_{↑}\equiv \gamma$. Additionally, the difference between energy points ↑ and ⇓ correspond to the difference between the two energy levels at which the topological changes already discussed occur. Furthermore, in the low-energy regime, entropy increases with positive slopes, while, in the high-energy regime, entropy decreases with negative slopes.

In contrast, pristine graphene γ=1 exhibits an entropy curve with a singular point (⇛) of slope change coinciding with the energy level associated with the topological transition between low- and high-energy regimes. As expected, for E≪1, the entropy curve fits the equation $S_{G}/k_{B}=2\ln\left(E\right)$ according to the equipartition principle [20,21]. Furthermore, the maximum entropy achieved by pristine graphene is lower compared to its doped counterparts, due to the increased symmetries of the spectrum in the absence of compositional variation.

On the other hand, entropy curves also exhibit inflection points that are directly associated with the behavior of other thermodynamic properties. Specifically, doped graphene displays two inflection points in the low-energy regime and only one inflection point in the high-energy regime. In comparison, pristine graphene has only one inflection point in each regime.

### 4.2. Temperature

Figure 3b depicts temperature T as a function of dimensionless energy E, obtained numerically from Equation (5). In the presence of a restricted energy spectrum, both positive and negative temperatures are evident [27,28,29,30], as illustrated in this figure. Additionally, the temperature is bounded, exhibiting global maxima and minima for all studied cases. These critical points coincide with the inflection points of the entropy curve. For doped graphene (1<γ<2), two critical points emerge in the low-energy regime, dividing the curve around the point ⇓ (refer to the inner panel in Figure 2b), due to the topological change that occurs in the low-energy spectrum as a result of the dopant. The energy point that divides the temperature curve is related to the effect of the dopant by the linear equation $T_{⇓}=-0.348\;\gamma +0.686$ for 1<γ<2 (with $R^{2}=0.999$). Moreover, it is crucial to emphasize that these points not only mark the topological transitions but also indicate the boundaries between positive and negative temperatures of the system. In this regime, the doped case reaches a higher temperature range compared to the pristine counterpart, directly attributed to the applied compositional variation ($T_{max}\propto \gamma$), and can be fitted with the quadratic relation $T_{max}=-0.289\gamma^{2}+1.286\gamma -0.603$, for 1<γ<2 with $R^{2}=0.999$. Moreover, for energies below at the point ⇓, temperature values demonstrate a linear behavior conforming to E~2T, consistent with the equipartition principle and the Dirac equation [20,21].

On the other hand, in the high-energy regime, negative temperatures (T<0) exists, and the doped graphene exhibits a critical point (global minimum) lower than the pristine graphene. This can be linked to the effect of the dopant using the equation $T_{min}=0.025\gamma^{2}-0.038\gamma -1.280$ with $R^{2}=0.999$ and for 1<γ<2. Subsequently, the complete temperature range attributed to the dopant can be expressed as $∆T=0.314\gamma^{2}-1.323\gamma -0.676$ for 1<γ<2, with $R^{2}=0.999$. Note that, for both regimes, there is no one-to-one correspondence between temperature and energy, suggesting a specific type of phase transition. Additionally, the temperature curves display discontinuities that align with the non-differentiable points on the entropy curve (at points ↑ and ⇛), potentially connected to the third law of thermodynamics [21].

In comparison, pristine graphene γ=1 exhibits only one critical point for each regime and has a smaller temperature range than doped graphene. For example, the temperature range for γ=1.9 is approximately 1.3 times greater than that of pristine graphene. Moreover, within the low-energy regime, temperature values follow a linear pattern, conforming to E~2T according to the equipartition principle and the Dirac equation [20,21].

### 4.3. Specific Heat

Figure 3c shows the relationship between specific heat C and dimensionless energy E, obtained through a numerical calculation using Equation (6). In general, it is observed that the points where ∂T/∂E=0 (see Figure 3b) are directly correlated with the divergences in specific heat. Furthermore, within these divergences, a finite region of negative specific heat (C<0) is generated. It is worth noting that this region coincides with the topological transition between the low- and high-energy regimes in the spectrum. Additionally, the energy domain $∆E_{C}$ of this region is strongly linked to the effect of the dopant, and, numerically, this domain increases as the γ value increases ($∆E_{C}\sim \gamma )$. Theoretically, these divergences arise from the fundamental thermodynamic identity [27,28,29,30]:

(8) $$T^{2}C\left(\frac{\partial^{2}S}{{\partial E}^{2}}\right)=-1$$

Thus, when the entropy exhibits inflection points at a finite temperature, the specific heat diverges. The graphs illustrate that, within the domain where C<0 (see Figure 3c), the second derivative of entropy is positive (see Figure 3a), indicating an association with a minimum of entropy, and serving as a signature of instability.

In the low-energy regime of doped graphene, Figure 3a reveals two divergences: the first around the energy points indicated by ⇓ and the second around the energy point indicated by ↑, exhibiting asymptotes at the critical points shown by the temperature curve (see Figure 3b). In contrast, the high-energy regime displays a single divergence, projected according to the same criterion mentioned earlier. These divergences are connected to topological changes on the surface of the energy spectrum, indicating significant topological transitions. Additionally, there are points C=0 that correspond to the inflection points of the entropy (see Figure 3a). Notably, these C=0 points align with T=0 (see Figure 2b), following the Nernst–Planck theorem [21]. In contrast to pristine graphene (γ=1), numerical calculations indicate a point C=0 associated with the entropy inflection point at E=1. Furthermore, two divergences occur at critical energy values, approximately at E~0.76 and E~1.36.

**Figure 4:**
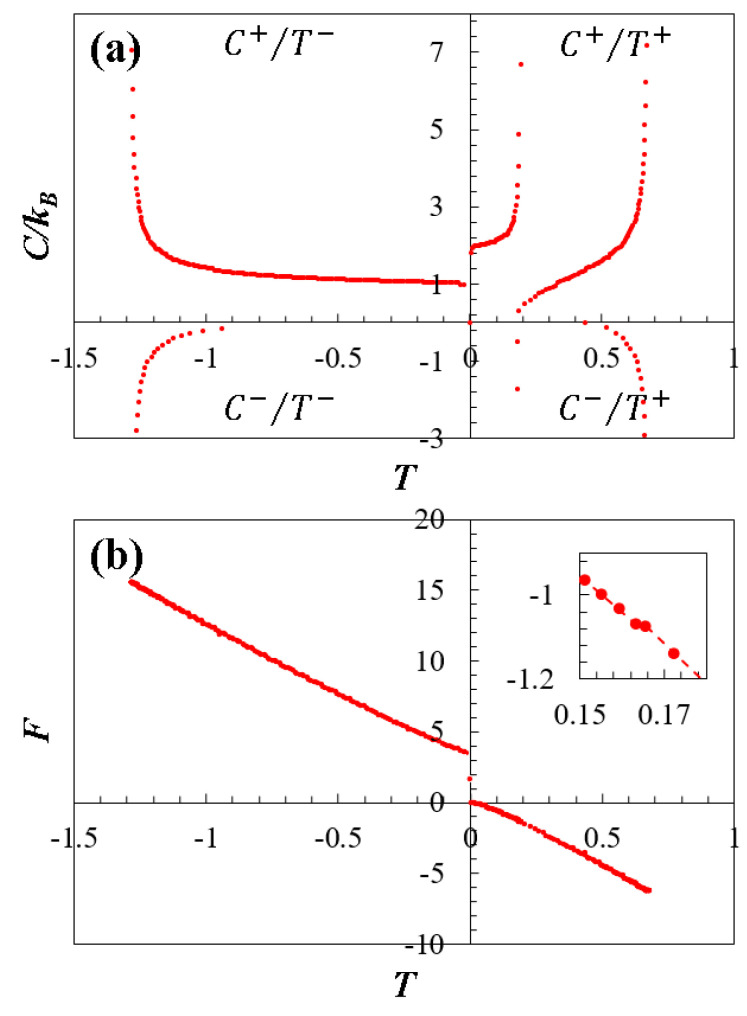
Thermodynamic behavior as a function of temperature for doped graphene (γ=1.5). (**a**) Specific heat C(T): The graph is divided into four quadrants that characterize the stabilities and instabilities of the system, designated as $C^{+}/T^{+}$, $C^{-}/T^{+}$, $C^{+}/T^{-}$, and $C^{-}/T^{-}$ (see their description in the text). (**b**) Helmholtz free energy F(T): The graph shows the discontinuities caused by the topological transition between the low- to high-energy regime. The inset highlights the topological transition in the low-energy regime.

Figure 4a illustrates the thermodynamically stable and unstable regions of doped graphene by depicting specific heat values as a function of temperature C(T). In this context, doped graphene with γ=1.5 serves as an example of its general thermodynamic behavior. This graph is divided into four quadrants for studying the thermodynamic characteristics. The $C^{+}/T^{+}$ quadrant, signaling stable behavior, exhibits positive $C^{+}$ values when $T^{+}$ is positive. Conversely, the $C^{-}/T^{+}$, $C^{+}/T^{-}$, and $C^{-}/T^{-}$ quadrants, where at least one parameter is in the negative range, indicate thermodynamically unstable behavior. Notably, the $C^{+}/T^{+}$ quadrant comprises two curves representing stability in the low-energy region (at $0<E<E_{⇓}$ and $E_{⇓}<E<E_{↑}$, respectively), while the unstable quadrants relate to the higher energy range ($E_{↑}<E<E_{max}$).

### 4.4. Helmholtz Free Energy

Figure 3d illustrates the thermodynamic behavior of the Helmholtz free energy F as a function of the dimensionless energy E, derived from numerical calculations utilizing Equation (7). As observed, the Helmholtz free energy exhibits behavior mirroring that of temperature (see Figure 3b), but opposite, as dictated by its relationship in Equation (7), and as theoretically expected. Notably, transitions at $E_{⇓}$ and $E_{↑}$ are also evident. For a better understanding of these transitions, Figure 4b presents the Helmholtz free energy as a function of temperature F(T), using γ=1.5 as example. This graph distinctly outlines the discontinuities caused by the topological transition between low- and high-energy regimes, directly associated with point $E_{↑}$ in Figure 3d. Moreover, the inner panel of Figure 3b highlights the topological transition in the low-energy regime at $E_{⇓}$, attributed to the merging of the Dirac cones as discussed earlier.

## 5. Discussion

**Figure 5:**
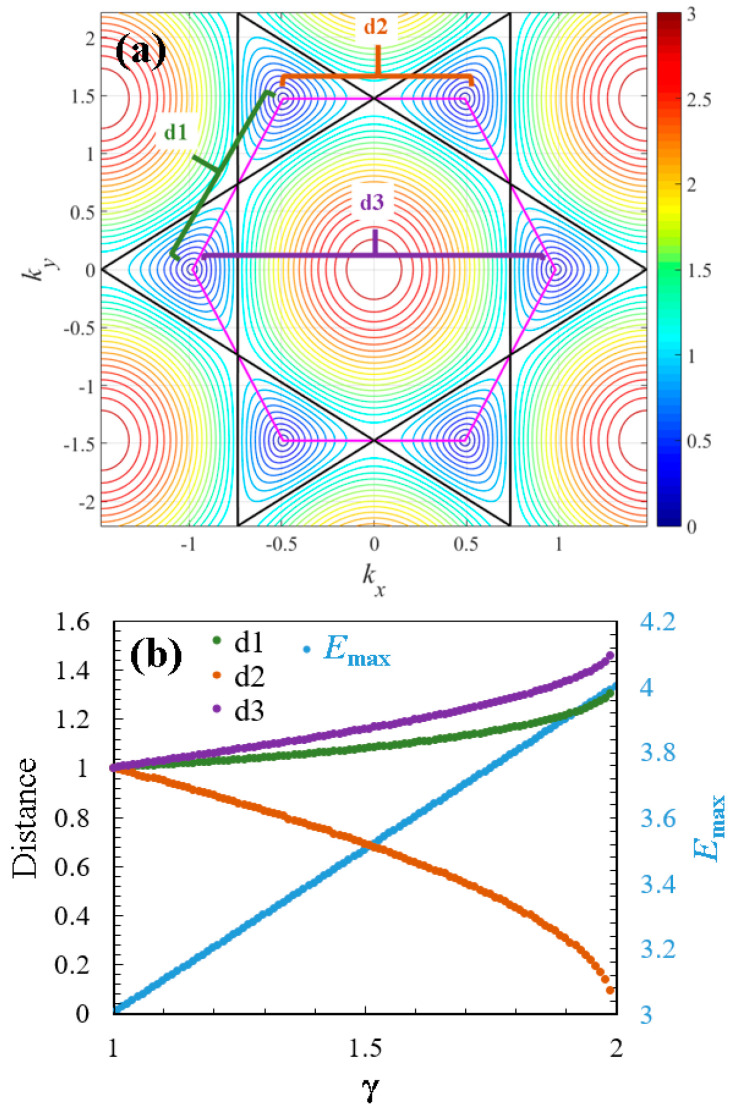
Topological changes caused by the heavy dopant atoms: (**a**) distances $d_{1}$, $d_{2}$, and $d_{3}$ between the centers of the Dirac cones; and (**b**) a double-axis plot representing the effect of dopant $\left(1<\gamma <2\right)$ on the distances $d_{1}$, $d_{2}$, and $d_{3}$ and the maximum energy $E_{max}$ of the graphene.

A topological transition induces changes in the topological properties of a material’s electronic structure in response to external parameters. In the case of doped graphene, this transition is driven by the effect of γ and involves changes in the connectivity of the Dirac cones or alterations in their topological invariants, which modify their electronic states. Figure 5 summarizes the effect of the dopant (for 1<γ<2) on the energy spectrum topology of graphene. In Figure 5a, distances $d_{1}$, $d_{2}$, and $d_{3}$ between the centers of the Dirac cones are illustrated. As mentioned previously, the compositional variation represented by γ causes a shift in the centers of the cones, leading to their merging, as shown in Figure 2b. In this context, the distance $d_{2}$ decreases when γ increases ($d_{2}\propto 1/\gamma$), while the distances $d_{1}$ and $d_{3}$ increase with γ ($d_{2},d_{3}\propto \gamma$). Another notable effect is the increase in the range of values contained in the energy surface of the spectrum. Figure 4b demonstrates how the maximum energy value increases with γ ($E_{max}\propto \gamma$). These topological changes induced by the dopant also affect the thermodynamic properties of the material.

The thermodynamic behavior of doped graphene 1<γ<2, as observed in Figure 3 and Figure 4, provides evidence of thermodynamic phase transitions directly associated with topological changes in the energy surface of its spectrum, induced by the effect of the dopant. Key findings include the influence of the dopant on Gibbs entropy, affecting the spectrum curve range $E_{max}\propto \gamma$ and maximum entropy $S_{G,max}\propto \gamma$. A distinct point at $E_{⇓}$ in the low-energy regime aligns with the merging of Dirac cones, indicating a topological change. Entropy curves exhibit different behaviors below and above $E_{⇓}$, with quasi-linear behavior observed beyond $E_{⇓}$ and inflection points linked to these topological changes. Moreover, critical points of temperature coincide with entropy inflection points. Doped graphene shows two critical points in the low-energy regime and one in the high-energy regime, all related to the same topological changes. Additionally, divergences in specific heat correlate with points where ∂T/∂E=0, and the C<0 region aligns with topological transitions. The C(T) graph identifies stable and unstable regions due to topological changes caused by the heavy dopant atoms. Furthermore, discontinuities in the F(T) graph provide evidence of these phenomena (see Figure 4).

Finally, we have developed a model that simulates the thermodynamic properties of heavily doped graphene based on its dispersion relation, enabling us to simply study the effects of various dopant atoms. Our work, in particular, aligns with hypothetical models of N/B-doped graphene as proposed by [16,22,24], or Si-doped graphene as studied by [23]. Additionally, our results are also consistent with other highly valued models in materials science, such as left-handed materials, where similar thermodynamic behaviors to those presented by our model have been observed, e.g., [31,32].

## 6. Conclusions

We have developed a model that simulates the thermodynamic properties of highly doped graphene through its dispersion relation, providing a straightforward approach to study the effects of different dopant atoms. Using a tight-binding model incorporating a parameter related to the effects of heavy dopants γ, we have explored how these dopants alter the electronic and thermodynamic properties of graphene. Our findings demonstrate that substitutional doping with heavy atoms significantly impacts the topology of graphene’s electronic structure, modifying Dirac cone positions, symmetry, and connectivity.

As an application of the model, we numerically simulate two types of graphene doped with heavy substitutional atoms in a hexagonal lattice, assuming γ=1.5 and γ=1.9 (as an upper bound in the model). By calculating the Gibbs entropy, temperature, specific heat, and Helmholtz free energy, we demonstrate that doped graphene within the range 1<γ<2 exhibits thermodynamic phase transitions linked to topological changes induced by heavy atoms. Notably, changes in the spectrum curve range ($E_{max}\propto \gamma$), the Gibbs entropy range, and maximum entropy ($S_{G,max}\propto \gamma$) highlight the influence of the dopant. Additionally, a distinct low-energy point $E_{⇓}$ marks the merging of Dirac cones, with inflection points in entropy and specific heat divergences that indicate stable and unstable thermodynamic regions. Discontinuities in the Helmholtz free energy further confirm these transitions.

Our results highlight the complex effects that heavy dopants have on the thermodynamic properties of graphene, laying a foundation for future experimental studies on doped graphene. They are consistent with prior research on hypothetical N/B-doped graphene [16,22,24] and Si-doped graphene [23]. Moreover, our model shows compatibility with other prominent materials science models, such as left-handed materials, where similar thermodynamic behaviors have been observed.

## Data Availability

The original contributions presented in the study are included in the article/Supplementary Material, further inquiries can be directed to the corresponding author.

## Supplementary Materials

The following supporting information can be downloaded at: https://www.mdpi.com/article/10.3390/e26121093/s1.
